# Generative artificial intelligence in public health: a framework for governance and systemic integration

**DOI:** 10.3389/fmed.2026.1614656

**Published:** 2026-06-11

**Authors:** Ruiye Yang, Mengqi Deng, Xiaoran Zheng, Yaoqi Deng, Junyi Jiang, Jinwei Miao

**Affiliations:** 1 Department of Gynecological Oncology, Beijing Obstetrics and Gynecology Hospital, Beijing Maternal and Child Health Care Hospital, Capital Medical University, Beijing, China; 2 Department of Gynecology and Obstetrics, Handan Fukang Hospital, Handan, Hebei, China; 3 Department of Educational Management, Nanchang University, Nanchang, Jiangxi, China; 4 State Key Laboratory of Medical Proteomics, National Center for Protein Sciences (Beijing), Beijing Institute of Life Omics, Beijing, China

**Keywords:** algorithmic bias, co-evolution, generative artificial intelligence, health equity, public health governance, Responsible Innovation

## Abstract

Generative artificial intelligence (GenAI) is poised to transform public health systems through its capacity for data synthesis and predictive modeling. This systematic review, analyzing 119 key studies, adopts a co-evolutionary lens to examine the dynamic interplay between GenAI advancements and public health system adaptation. We demonstrate that the effective integration of GenAI is fundamentally constrained by a system’s infrastructural, institutional, and human resource maturity. Our analysis, grounded in the theory of Responsible Innovation, identifies three interconnected governance domains–technical transparency, institutional accountability, and ethical equity–that frame the core challenges. We subsequently propose a three-layer governance framework to navigate these issues, emphasizing that trustworthy AI ecosystems require more than technical excellence; they demand institutional foresight, inclusive governance, and a steadfast commitment to equitable, human-centered health futures.

## 1 Introduction

**FIGURE 1:**
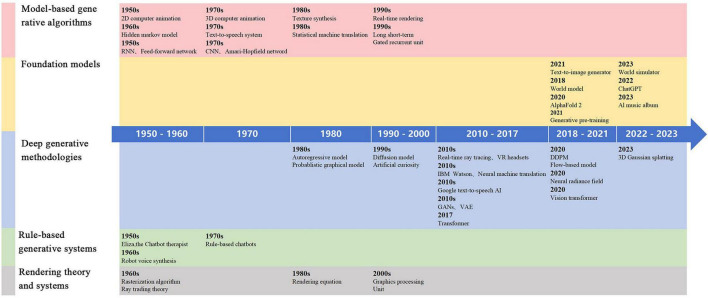
Timeline of the development of GAI methods and applications. The figure traces the evolution from rule-based generative systems (1950s–1960s) and deep generative methodologies (2010s) to foundation models (2018–2023). Key milestones include Eliza, GANs, VAE, Transformer, diffusion models, AlphaFold 2, ChatGPT, and 3D Gaussian splatting.

The technological evolution of generative artificial intelligence (GenAI) has progressed through four distinct paradigms (Figure 1): from rule-based systems to probability-driven models, followed by statistical deep generative models, and now multimodal foundation models like GPT-4 and Google Gemini (1). Concurrently, public health systems have undergone their own transformation, expanding from a foundational focus on sanitation and infectious disease control to encompass chronic disease prevention, health promotion, and global health security (2).

This paper positions itself as a conceptual and policy-oriented analysis aimed to contribute new ways of thinking about the transformative impact of GenAI on public health systems. Rather than presenting empirical findings, we synthesize existing research to develop a novel governance framework that addresses the critical tensions at this intersection.

**FIGURE 2:**
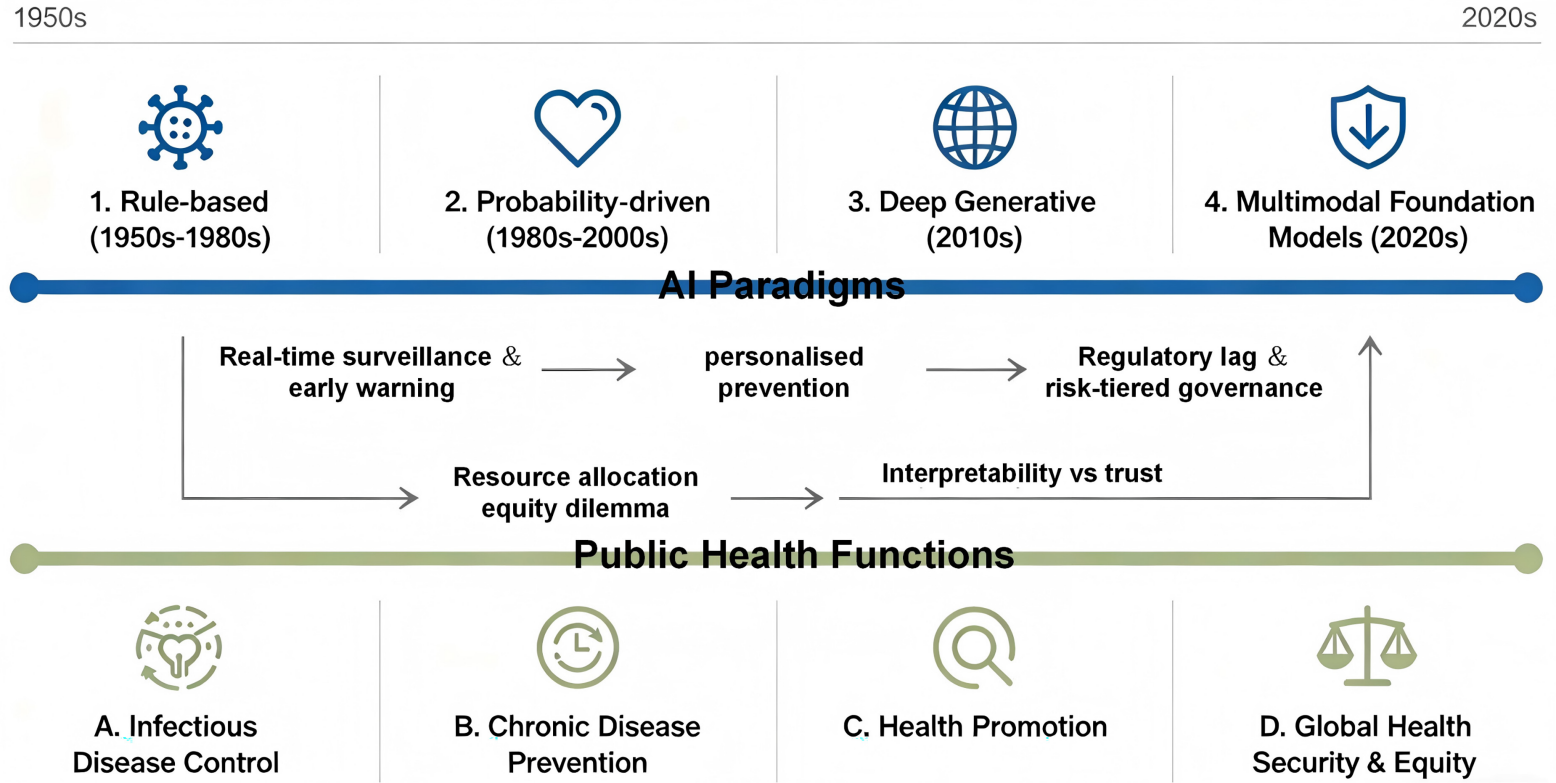
Co-evolutionary pathways between generative AI paradigms and public health system functions. Four AI paradigms (rule-based, probability-driven, deep generative, multimodal foundation) are aligned with four public health functions (infectious disease control, chronic disease prevention, health promotion, global health security and equity). Bidirectional arrows highlight key interaction points: real-time surveillance, personalized prevention, regulatory lag, resource allocation bias, and interpretability-trust trade-offs.

The central thesis of this paper is that the relationship between GenAI and public health is best understood as a co-evolutionary process, where technological advancements and system adaptations mutually influence each other over time. As illustrated in Figure 2, the co-evolution between successive AI paradigms and public health system functions presents distinct challenges and opportunities for public health functions, requiring corresponding adaptations in system governance and practice. The critical question is: How is GenAI being taken up by public health systems, and which core functions are most susceptible to transformation?

Generative artificial intelligence tools, characterized by their capacity to synthesize unstructured, multimodal data and generate novel insights, hold significant promise for revolutionizing public health functions such as real-time surveillance, predictive modeling, and personalized prevention strategies. However, the realization of this potential is not automatic. It is critically mediated by the maturity and adaptive capacity of the public health system itself–its data infrastructure, regulatory agility, and workforce competency (3, 4).

This review aims to: (1) analyze the transformative impact of GenAI on key public health functions, (2) identify the core governance challenges arising from the tension between technological innovation and system readiness, and (3) propose a multidimensional governance framework, grounded in established theory, to guide the development of trustworthy and equitable GenAI applications. The remainder of this paper is organized as follows: Section 2 outlines our review methodology; Section 3 establishes our conceptual perspective; Section 4 analyzes GenAI’s impact on public health; Section 5 discusses governance challenges; Section 6 outlines our theorized governance framework; and Section 7 presents concrete implementation pathways.

## 2 Review methodology

This review was conducted following a systematic literature search and analysis protocol to ensure comprehensiveness and reproducibility. Electronic databases, primarily PubMed/MEDLINE, were searched for articles published between January 2015 and March 2026. The search employed a combination of controlled vocabulary terms (MeSH) and free-text keywords, including but not limited to: “generative artificial intelligence,” “large language models,” “ChatGPT,” “public health,” “healthcare,” “clinical medicine,” “governance,” “ethics,” “regulation,” and “implementation.” Additional records were identified through citation tracking of key systematic reviews and expert consultation. Our selection criteria prioritized peer-reviewed articles, seminal pre-prints, and influential policy reports from international organizations.

A total of 1,030 records were initially identified through PubMed/MEDLINE (*n* = 1,002) and other sources including citation tracking of key systematic reviews and expert consultation (*n* = 28). After removing 120 duplicates, 910 records were screened by title and abstract. Of these, 650 records were excluded as not directly relevant to generative AI in public health contexts or due to inappropriate study design. The remaining 260 full-text articles were assessed for eligibility; 141 were excluded for reasons including no governance focus, irrelevant study design, full text not accessible, or insufficient data for synthesis. Finally, 119 studies met the inclusion criteria and were included in the thematic synthesis (Supplementary Table 1). Literature screening and data management were supported by Microsoft Excel (for coding and theme extraction) and Mendeley (for duplicate removal and reference organization). No dedicated qualitative software (e.g., NVivo, ATLAS.ti) was used due to the manageable scale of 119 included studies. All coding and theme development were performed independently by three reviewers (R.Y., M.D., X.Z.) with disagreements resolved through team discussion. The analytical approach was thematic, focusing on identifying, analyzing, and structuring core governance challenges and proposed solutions across technical, institutional, and ethical dimensions.

To ensure transparency in how the three governance dimensions emerged from the literature, we performed a deductive thematic mapping of the 119 included studies. Each study was independently coded by two reviewers (R.Y. and M.D.) for the presence of themes related to technical transparency (e.g., interpretability, explainability, data provenance), institutional accountability (e.g., regulatory frameworks, governance structures, liability), and ethical equity (e.g., algorithmic bias, health disparities, benefit distribution). Studies could be assigned to one or more dimensions. The resulting mapping is provided in Supplementary Table 1, which includes an additional column (“Relevant Dimension(s)”) indicating the dimension(s) each study supports.

**FIGURE 3:**
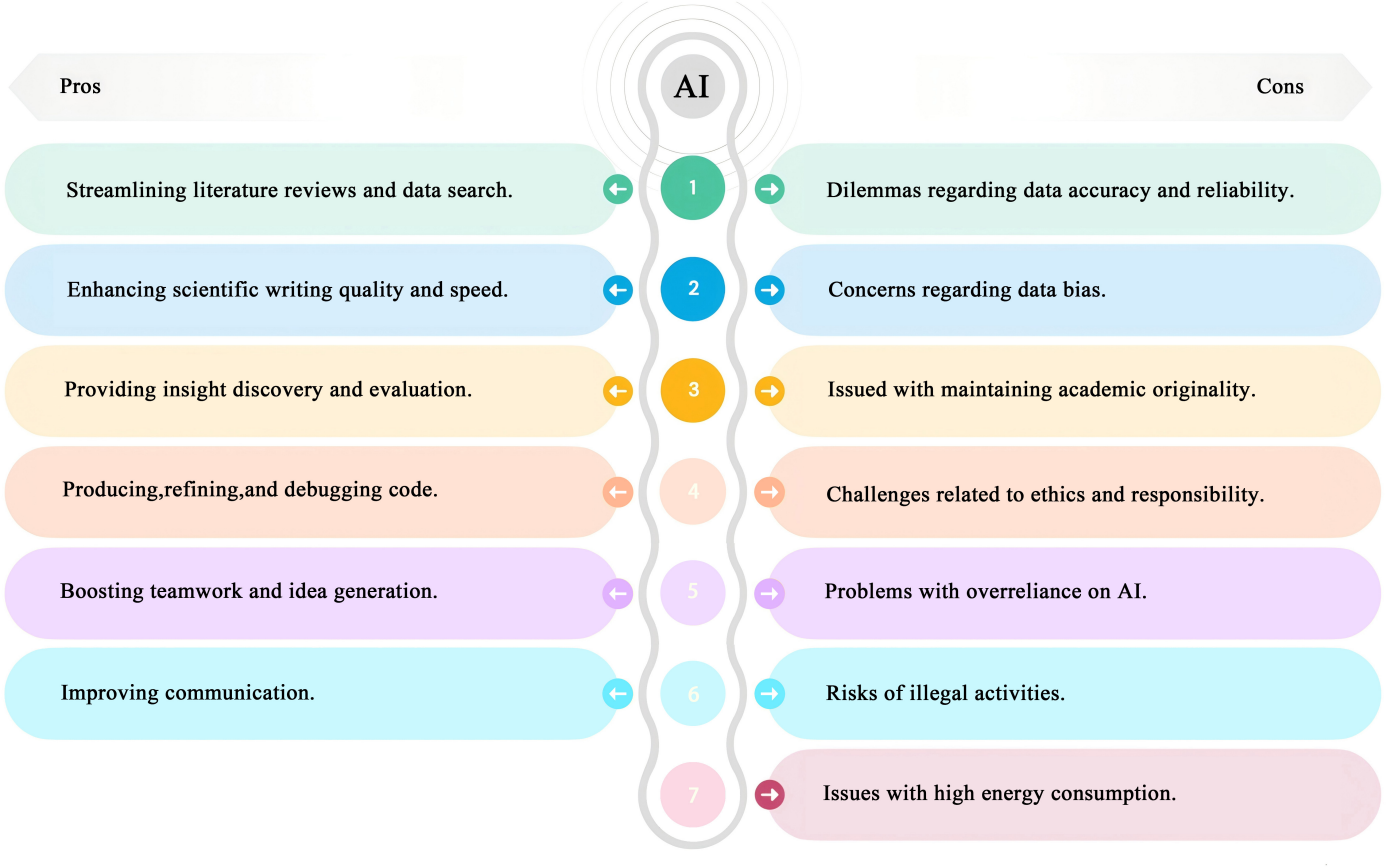
Pros and cons of generative AI impact on the scientific community. Positive aspects include streamlining literature reviews, enhancing writing quality, insight discovery, code debugging, teamwork, and communication. Negative aspects include concerns over data accuracy and reliability, data bias, loss of academic originality, ethical and responsibility challenges, overreliance, illegal activities, and high energy consumption.

**FIGURE 4:**
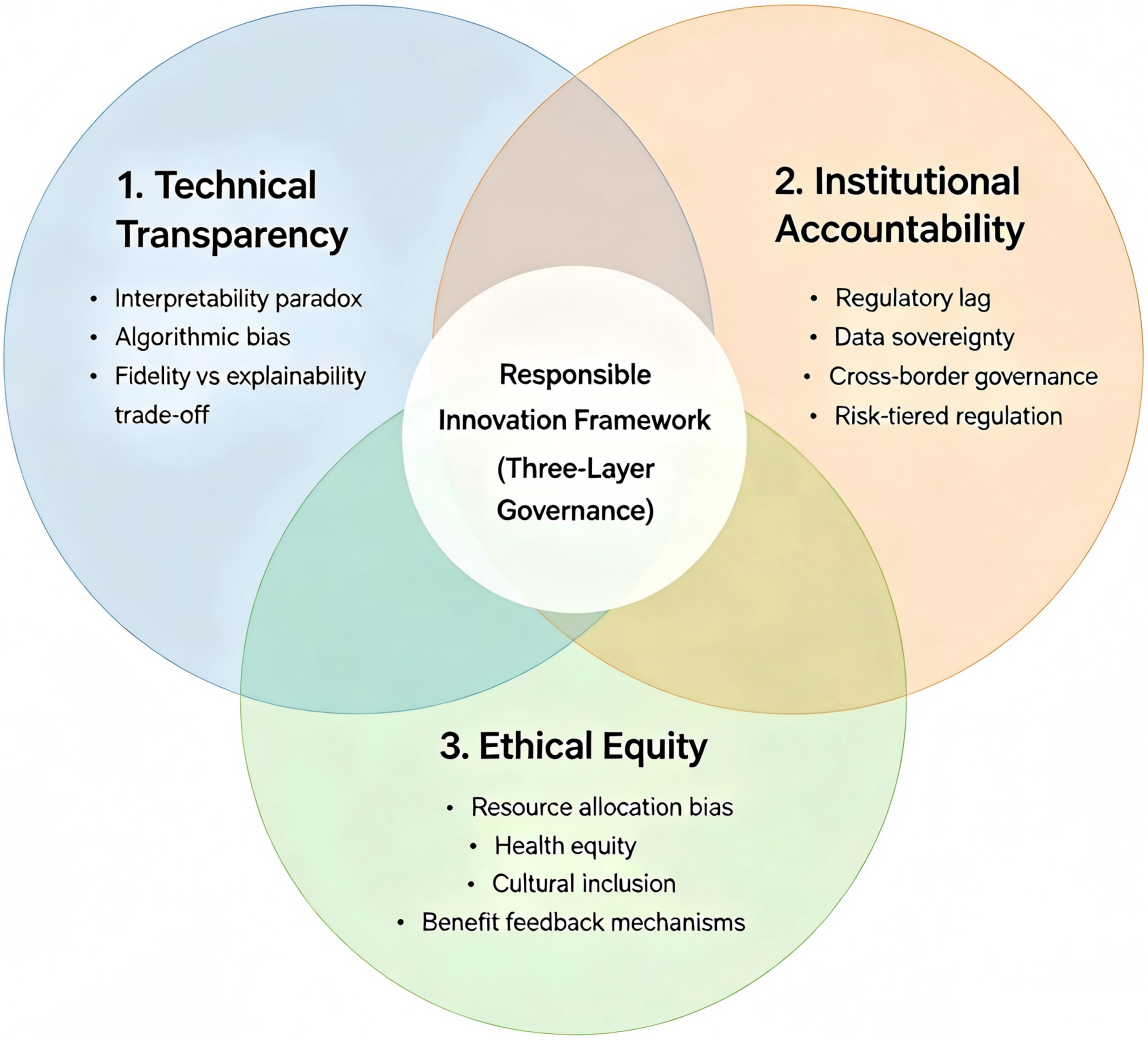
Three interconnected governance domains identified from the systematic literature analysis. (1) Technical transparency – interpretability paradox, algorithmic bias, fidelity-explainability trade-off. (2) Institutional accountability – regulatory lag, data sovereignty, cross-border governance, risk-tiered regulation. (3) Ethical equity – resource allocation bias, health equity, cultural inclusion, benefit feedback mechanisms.

As visualized in Figures 3, 4, the literature analysis revealed three interconnected domains of challenge and opportunity–technical transparency, institutional accountability, and ethical equity–that directly informed the development of our three-layer governance framework. The selected papers provided the evidentiary basis for identifying the specific issues addressed in Sections 4–7. Figure 3 summarizes the discrete pros and cons reported in the literature (descriptive inventory). Through thematic synthesis, these were clustered into the three higher-order governance domains shown in Figure 4 (conceptual output). Thus, Figure 4 represents the analytical result derived from the raw evidence presented in Figure 3.

## 3 Conceptual perspective: GenAI and public health system appropriation

To ground our analysis, we situate it within two complementary theoretical frameworks: co-evolutionary theory from complexity and socio-technical systems literature (2, 3, 5), and the theory of Responsible Innovation (RRI) (6).

The concept of co-evolution helps explain the dynamic, reciprocal relationship between technological innovation and health system adaptation. As Geels (3) argues, technological change does not simply determine social outcomes; rather, technologies and social systems shape each other through complex feedback loops. In the context of public health, this means that GenAI’s impact will be determined not only by its technical capabilities but by how these capabilities interact with existing system structures, professional practices, and institutional arrangements (5).

This co-evolutionary perspective is enhanced by the principles of Responsible Innovation, which provides a normative framework for guiding this interactive process. RRI emphasizes four dimensions (6): Anticipation: Systematically considering potential impacts and implications of innovation; Reflexivity: Reflecting on underlying assumptions, values, and uncertainties; Inclusion: Opening up innovation processes to diverse stakeholders; Responsiveness: Using this knowledge to adapt innovation trajectories.

Together, these theoretical perspectives inform our core proposition: the appropriation of GenAI advancements depends on the public health system’s maturity level across several dimensions, and this appropriation process should be guided by RRI principles. Our subsequent analysis applies this integrated perspective to examine both the technological possibilities and the necessary systemic adaptations across different public health functions.

## 4 The co-evolutionary transformation of public health functions

This co-evolutionary dynamic, conceptualized in Figure 2, informs the following analysis of specific public health functions. Figure 2 presents a historical co-evolutionary timeline of public health functions–from infectious disease control (A) to chronic disease prevention (B), health promotion (C), and global health security and equity (D). In the current analysis, we focus on two cross-cutting dynamics that are most prominently reshaped by GenAI and that span across these functions: surveillance/precision prevention (Section 4.1) and resource allocation/equity dilemmas (Section 4.2). Insights related to chronic disease prevention and health promotion are woven throughout the discussion where relevant. We analyze how the integration of advanced AI tools necessitates corresponding changes in system operations and governance approaches.

### 4.1 Enhancing surveillance and precision prevention

Generative artificial intelligence is reshaping public health surveillance by integrating multi-source, heterogeneous data. In infectious disease early warning, multimodal models have demonstrated significant utility. For instance, a model integrating flight passenger data, climate variables, and local case reports achieved an 85% prediction accuracy for dengue fever outbreaks in Southeast Asia 3 weeks in advance, demonstrating a tangible improvement in the timeliness of epidemic response, a core public health function (7). Furthermore, by synthesizing geospatial data, GenAI reveals unique value in disease transmission modeling and environmental health risk assessment, overcoming the limitations of traditional, siloed data acquisition (8). For example, deep learning-based heat maps are now capable of identifying socio-ecological hotspots for zoonotic spillover events that were previously unidentifiable. While this subsection focuses on infectious disease control, similar GenAI applications are emerging for chronic disease prevention (e.g., personalized risk scoring for diabetes and cardiovascular diseases) and for health promotion through early warning and personalized risk communication.

### 4.2 Reconfiguring resource allocation and unveiling equity dilemmas

A fundamental tension exists between algorithmic efficiency prioritization and equitable outcomes in AI-based allocation systems. Studies indicate that algorithms trained on historical data can amplify existing disparities; one investigation found a 35% under-allocation of diagnostic resources to predominantly Black ZIP codes compared to their white counterparts with identical clinical risk scores (9). This highlights the risk of automating and scaling institutional discrimination.

Conversely, Geospatial Foundation Models (GFMs) combined with GenAI present a powerful solution for optimizing emergency resource allocation. These systems can synthesize real-time open-source intelligence (OSINT) and satellite imagery to produce dynamic, regionalized disaster prevention and control plans. Such integrations have been piloted to guide flood response and medical supply chain logistics during natural disasters in South Asia, showcasing a practical application for enhancing system resilience (10, 11). Beyond resource allocation, these equity dilemmas also affect chronic disease prevention (e.g., unequal distribution of screening resources) and health promotion (e.g., GenAI-driven chatbots disproportionately benefiting digitally literate populations).

## 5 Core governance challenges at the system-technology interface

This section explores the governance tensions that emerge from the uneven pace of change between technological innovation and institutional adaptation. The challenges identified here directly inform the governance framework developed in Section “6 A Responsible Innovation framework for GenAI governance.”

### 5.1 Institutional lag and the evolving regulatory landscape

Contemporary healthcare data governance frameworks confront dual challenges: early-stage data collection protocols inadequately regulate AI-driven secondary data mining, while cross-border data flows create risks of data sovereignty erosion. Although AI-generated synthetic data can mitigate privacy concerns, its generation process may inadvertently retain sensitive features from source datasets, necessitating novel ethical review mechanisms (12).

The regulatory landscape is evolving to meet these challenges. The European Union’s AI Act pioneers a risk-based approach, classifying most medical AI as “high-risk” and mandating strict *ex ante* conformity assessments and fundamental rights impact evaluations (13). In contrast, the U.S. FDA’s approach for AI/ML-based Software as a Medical Device (SaMD) emphasizes a predetermined change control plan, facilitating iterative updates to approved algorithms (14). The World Health Organization (WHO), meanwhile, provides guiding ethical principles that prioritize equitable access and stakeholder participation, offering a normative compass rather than binding law (15). Juxtaposing these frameworks reveals a critical tension: the EU’s comprehensive, precautionary model versus the FDA’s agile, product-centric pathway, highlighting the varying levels of institutional maturity required for effective governance.

### 5.2 Technical opacity and the interpretability paradox

Liability assignment for AI-driven medical errors remains profoundly challenging. It is often impossible to distinguish between data prejudice, model construction defects, and operational failures when a diagnostic error occurs. While Explainable AI (XAI) tools have improved, they often provide only a limited, partial view of the model’s internal reasoning, a phenomenon sometimes termed “the illusion of explanation” (16). A trade-off persists between interpretability and diagnostic fidelity in complex clinical settings like cardiology and genetic testing (17, 18), underscoring that legitimacy and trust require more than just high technical transparency scores (19).

## 6 A Responsible Innovation framework for GenAI governance

These three domains, summarized in Figure 4, map onto the three layers of our governance framework: technical transparency informs the technological layer, institutional accountability grounds the institutional layer, and ethical equity shapes the ethical layer. Building on the conceptual framework established in Section “3 Conceptual perspective: GenAI and public health system appropriation” and the challenges identified in Sections 4–5, this section develops a comprehensive governance approach grounded in Responsible Innovation principles. The framework addresses the co-evolutionary dynamics through three interconnected layers.

Our three-layer governance framework is substantively grounded in the theory of Responsible Innovation (RRI), which emphasizes four dimensions: anticipation, reflexivity, inclusion, and responsiveness (20). This theoretical foundation provides a “fil rouge” that connects our governance prescriptions to a broader scholarly discourse on managing emerging technologies.

### 6.1 Technological layer: interpretability and adaptive design

Dynamic optimization mechanisms and interpretability tools are crucial for fostering reflexivity. In health cartography, integrating XAI with geographic information systems allows researchers to trace the spatial decision logic of disease prediction models. For instance, attention mechanism visualizations facilitate the analytical weighting of environmental risk factors, such as pinpointing the specific contribution of pollution levels or population density to an outbreak prediction (21). This not only enhances technical transparency but is also a cornerstone for building clinician-patient trust.

### 6.2 Institutional layer: collaborative and risk-tiered governance

Institutional maturity is built through collaborative models that embody inclusion and responsiveness. Regional medical data platforms employing privacy-preserving computation technologies like federated learning have established secure data-sharing paradigms, offering novel solutions to data silo challenges while safeguarding data sovereignty (22). Furthermore, risk-tiered regulatory systems, which set scenario-specific control standards, exemplify the evolution of governance from one-size-fits-all approaches to precision-oriented methodologies, allowing for more efficient and targeted oversight.

### 6.3 Ethical layer: value alignment and equitable benefit distribution

Embedding localized medical cultural characteristics into AI training datasets significantly improves technological acceptability and mitigates cultural conflict. This is an act of ethical inclusion. Establishing technological dividend feedback mechanisms, where efficiency gains from AI are strategically reinvested into primary healthcare infrastructure, provides an operational pathway for alleviating health inequities and ensuring that benefits are distributed justly (23).

The convergence of these three layers is highly context-dependent. In countries with strong centralized health systems, institutional foresight might be more readily coordinated. In contrast, in decentralized or resource-constrained systems, technological adaptations and community-level ethical interventions may be the primary levers for improvement. Evidence from specific national contexts, such as China’s integration of AI into primary care screenings for diabetic retinopathy, demonstrates that convergence is possible but requires significant, sustained investment in public health infrastructure and workforce training–directly illustrating the role of systemic maturity (24).

## 7 Implementation pathways for trustworthy AI ecosystems

This section translates the research findings from our analysis into concrete implementation strategies. The pathways outlined here leverage the co-evolutionary perspective and RRI principles to inform practical approaches for developing trustworthy AI ecosystems in public health.

### 7.1 Development and application of intelligent regulatory tools (RegTech)

The advancement of regulatory technology (RegTech) enables real-time monitoring of AI system risks. Algorithmic impact assessment (AIA) tools are demonstrating early-warning value in policy experimentation. Building on the pharmaceutical-inspired regulation framework proposed by scholars, future exploration should focus on developing tools like RegulatoryGPT to assist policymaking through dynamic analysis of clinical data for systemic risk prediction (25). Furthermore, generative AI can be used to construct multi-agent systems that simulate ethical conflicts in public health decision-making, providing valuable stress-testing environments for policy formulation before real-world deployment.

### 7.2 Operationalizing transnational and participatory governance

Building trustworthy AI ecosystems requires concrete mechanisms for transnational coordination and grassroots inclusion. The vision of “transnational governance” can be operationalized through models akin to the International Panel on Climate Change (IPCC), establishing a global scientific consensus on AI risks and benefits, or by strengthening mutual recognition agreements (MRAs) for AI system certifications between major regulatory jurisdictions (26).

To move beyond tokenistic “participatory design,” institutions can adopt proven models such as “Citizens” Assemblies’ for deliberating on local AI priorities and “AI Ethics Auditing Juries” that include community representatives in the oversight of algorithmic systems (27). These mechanisms ensure that governance is not only top-down but also bottom-up, embedding societal values directly into the technological fabric and enhancing the legitimacy of AI systems.

### 7.3 Practical implications for different healthcare actors

The three-layer framework offers specific entry points across the health system. Policymakers and regulators should focus on the institutional layer – risk-tiered approvals, algorithmic impact assessments, and mutual recognition agreements. Healthcare administrators can use the technological layer (explainable AI, training) and the institutional layer (liability protocols, patient feedback). Clinicians can draw on the technological layer for interpretable tools and the ethical layer for equitable access. Developers should build in technical transparency (model cards, audit trails) and ethical equity (bias mitigation) from the start. Patients and communities have a role in the ethical layer through participatory governance (citizens’ assemblies, ethics juries) and in holding institutions accountable. Each layer provides a distinct but interconnected lever for action, and implementation requires coordination across these actors.

## 8 Conclusion

Generative AI presents a profound opportunity for a paradigm shift in public health, offering powerful new tools for protecting and promoting population health. However, our analysis confirms that the benefits of GenAI are not guaranteed; they are contingent on strengthening the core capacities of public health systems. The tensions between efficiency and equity, innovation and safety, are systemic and must be addressed as such.

The three-layer governance framework proposed herein, grounded in Responsible Innovation, provides a roadmap for navigating this complex landscape. Future priorities must include the development of multidimensional evaluation metrics that integrate clinical efficacy, equity, and societal impact; the establishment of agile, transnational governance models to mitigate data colonialism; and the genuine institutionalization of participatory design. Ultimately, trustworthy medical AI demands the co-evolution of technology and system maturity. Its success will be defined not by technical prowess alone, but by its sustained contribution to equitable health outcomes and its reinforcement of a more resilient, human-centered public health system for the future.

## Supplementary material

Supplementary Table 1 Characteristics of the included systematic reviews.
