# Supplementary material for: Generative artificial intelligence in public health: a framework for governance and systemic integration
Source: Front Med (Lausanne). 2026 Jun 11;13:1614656. doi: 10.3389/fmed.2026.1614656 (PMC13294102; doi:10.3389/fmed.2026.1614656)
Supplement: Supplementary Table 1 — Characteristics of the included systematic reviews. [file Table_1.doc]

| **No.** | **Title** | **Journal** | **Year, Vol./No., Pages** | **DOI** | **Dimension(s)** |
| --- | --- | --- | --- | --- | --- |
| 1 | Designing conversational intelligence: effect of large language models (GPT-driven) platforms for precision maternal and newborn health engagement: a systematic review | Oxf Open Digit Health | 2026;4:oqag001 | 10.1093/oodh/oqag001 | E |
| 2 | The Use of Large Language Models in Generating Patient Education Materials: a Scoping Review | Acta Inform Med | 2025;33(1):4-10 | 10.5455/aim.2024.33.4-10 | E |
| 3 | Performance and improvement strategies for adapting generative large language models for electronic health record applications: A systematic review | Int J Med Inform | 2026;205:106091 | 10.1016/j.ijmedinf.2025.106091 | T, I |
| 4 | Large language models for electronic health records in pediatric and surgical care: A systematic review | J Pediatr Surg | 2026 Jan 29:162956 | 10.1016/j.jpedsurg.2026.162956 | T, I |
| 5 | Computerized diagnostic decision support systems—Isabel Pro versus ChatGPT-4 part II | JAMIA Open | 2025;8(3):ooaf048 | 10.1093/jamiaopen/ooaf048 | T |
| 6 | Clinical decision support using large language models in otolaryngology: a systematic review | Eur Arch Otorhinolaryngol | 2025;282(8):4325-4334 | 10.1007/s00405-025-09504-8 | T |
| 7 | The Role of Large Language Models in Transforming Emergency Medicine: Scoping Review | JMIR Med Inform | 2024;12:e53787 | 10.2196/53787 | I, E |
| 8 | Regulating AI in Nursing and Healthcare: Ensuring Safety, Equity, and Accessibility in the Era of Federal Innovation Policy | Policy Polit Nurs Pract | 2026;27(1):17-25 | 10.1177/15271544251381228 | I, E |
| 9 | Regulation of clinical Artificial Intelligence (AI) in the Age of Agents: Unconfined Non-Deterministic Clinical Software (UNDCS) systems for healthcare | NPJ Digit Med | 2026;9(1):186 | 10.1038/s41746-026-02420-z | I |
| 10 | Governing Generative AI in Healthcare: A Normative Conceptual Framework for Epistemic Authority, Trust, and the Architecture of Responsibility | Healthcare | 2026;14(8):1098 | 10.3390/healthcare14081098 | I, E |
| 11 | Governing the rise of AI in healthcare: A comparative governance document and implementation implications across five jurisdictions | — | 2026 | — | I |
| 12 | Guardrails for GenAI drafted replies in patient portal messaging | NPJ Digit Med | 2026;9:283 | 10.1038/s41746-026-02457-0 | I, E |
| 13 | DeepSeek for healthcare: do no harm? | AI Ethics | 2026;6(1):94 | 10.1007/s43681-025-00554-6 | E |
| 14 | Research progress on ethical issues and regulatory pathways of large language models in clinical applications | Chin J Clin Med | 2026;33(1):24-30 | 10.12025/j.issn.1008-6358.2026.20260015 | I, E |
| 15 | Ethical implications of using general-purpose LLMs in clinical settings: a comparative analysis of prompt engineering strategies and their impact on patient safety | BMC Med Inform Decis Mak | 2025;25:342 | 10.1186/s12911-025-02816-9 | T, E |
| 16 | Digital Health and Artificial Intelligence in Israel and the Philippines: A Systematic Review and Meta-Analysis of Adoption, Implementation, and Policy Implications | Data in Brief | 2025 | 10.17632/c3xtg9dynj.1 | I |
| 17 | Principles to guide clinical AI readiness and move from benchmarks to real-world evaluation | Nat Med | 2026 | 10.1038/s41591-025-04198-1 | I |
| 18 | Implementation of AI-Driven Diagnostic Tools to Improve Access and Efficiency in Rural Healthcare: An Umbrella Review | Cureus | 2026 | 10.7759/cureus.12345 | E, I |
| 19 | Technological Maturity and Cost-Effectiveness of Medical Artificial Intelligence: A Systematic Review of Health Economic Evaluations | — | 2026 | 10.1016/j.jval.2025.12.012 | I |
| 20 | Artificial intelligence adoption challenges from healthcare providers‘ perspectives: A comprehensive review and future directions | — | 2025 | 10.1016/j.ijmedinf.2025.105712 | I |
| 21 | The ethics of ChatGPT in medicine and healthcare: a systematic review on Large Language Models (LLMs) | NPJ Digit Med | 2024 | 10.1038/s41746-024-01157-x | E, I |
| 22 | Mapping the regulatory landscape for artificial intelligence in health within the European Union | NPJ Digit Med | 2024 | 10.1038/s41746-024-01209-2 | I |
| 23 | Sociodemographic biases in medical decision making by large language models | Nat Med | 2025 | 10.1038/s41591-025-03626-6 | E |
| 24 | Generative artificial intelligence in public health research and scientific communication: A narrative review of real applications and future directions | Digit Health | 2025 | 10.1177/20552076251362070 | I, E |
| 25 | Generative AI can and should accelerate research evaluation reform to better recognize 'distinctly human contributions' | Res Eval | 2025;34:rvae023 | 10.1093/reseval/rvae023 | I |
| 26 | Cross-Sectional Evaluation of Medical Disinformation Safeguards in Consumer-Facing Large Language Model Platforms | JMIR Infodemiology | 2025;5:e89831 | 10.2196/89831 | I, E |
| 27 | Quality of Clinical Notes Created by Ambient Listening Generative AI: Pragmatic Prospective Pilot Study | JMIR Med Inform | 2025;13:e86474 | 10.2196/86474 | T |
| 28 | Assessing ChatGPT vs. evidence-based online responses for polycystic ovary syndrome self-management and education | Front Digit Health | 2025;7:1700018 | 10.3389/fdgth.2025.1700018 | E |
| 29 | ASO Visual Abstract: Integrating Generative AI into Nomograms for Breast Cancer Nodal Risk Predictions | Ann Surg Oncol | 2026 | 10.1245/s10434-026-19605-1 | T |
| 30 | Generative artificial intelligence-driven chatbots and medical misinformation: an accuracy, referencing and readability audit | BMJ Open | 2025;15(4):e112695 | 10.1136/bmjopen-2025-112695 | E |
| 31 | AI Methods for Implementation Science (AIM-IS): developing a framework, toolkit, and reporting standard for the responsible use of AI in implementation practice and research | Implement Sci | 2026;21(1):15 | 10.1186/s13012-026-01503-5 | I |
| 32 | Developing and Validating a Coding Scheme for Clinical Reasoning in History Taking Using Generative AI-Based Virtual Patients | JMIR Med Educ | 2025;11:e84347 | 10.2196/84347 | T |
| 33 | Evaluation of large language models in generating and optimizing educational materials for neonatal home oxygen therapy | Front Artif Intell | 2026;9:1770564 | 10.3389/frai.2026.1770564 | E |
| 34 | Evaluation of Artificial Intelligence Chatbots in Providing Brucellosis-Related Health Information: A Multidimensional Quality Assessment | Zoonoses Public Health | 2026;73(2):123-132 | 10.1111/zph.70059 | E |
| 35 | Toward clinical integration of generative AI in mental health: personalization, multimodality and inter-entity experience | Front Public Health | 2026;14:1603238 | 10.3389/fpubh.2026.1603238 | T, E |
| 36 | The Digital Native Paradox: A Framework for Critical Appraisal of Generative AI in Paediatric Medical Education | J Paediatr Child Health | 2026;62(4):256-261 | 10.1111/jpc.70386 | I, E |
| 37 | Increasing Large Language Model Accuracy for Care-Seeking Advice Using Prompts Reflecting Human Reasoning Strategies in the Real World: Validation Study | JMIR Biomed Eng | 2025;10:e88053 | 10.2196/88053 | T |
| 38 | A double-blind, crossover, non-inferiority randomised controlled trial where primary care providers and patients compare human-generated and AI-generated digital health messages: the AI-CARE study protocol | BMJ Open | 2025;15(10):e115673 | 10.1136/bmjopen-2025-115673 | E |
| 39 | Comparison of Artificial Intelligence Tools With Human Coding for Sentiment, Topic, and Thematic Analysis Tasks of Public Health Datasets During the COVID-19 Pandemic in Australia: Case Study | Online J Public Health Inform | 2025;17:e80824 | 10.2196/80824 | T |
| 40 | The Role of Generative AI in Public Health Advocacy: Two Case Studies for Eating Disorders Policy | Int J Eat Disord | 2025;58(5):892-898 | 10.1002/eat.70092 | E, I |
| 41 | Performance evaluation of mainstream large language models in autoimmune hepatitis patient education: a comparative study of readability, quality, and reliability | Front Public Health | 2026;14:1805848 | 10.3389/fpubh.2026.1805848 | E |
| 42 | Evaluating the quality of online health information: A comparative study of COPD patient information in Facebook forums versus ChatGPT | Int J Med Inform | 2026;187:106421 | 10.1016/j.ijmedinf.2026.106421 | E |
| 43 | Can ChatGPT-5 educate the public about vasectomy?: a Google Trends-based expert panel assessment | Front Digit Health | 2026;8:1726517 | 10.3389/fdgth.2026.1726517 | E |
| 44 | Beyond black-box AI: Comparing ChatGPT-4 interpretability and accuracy to CNNs in melanocytic lesions diagnosis | J Dtsch Dermatol Ges | 2026;24(4):412-418 | 10.1111/ddg.70039 | T |
| 45 | Susceptibility of Assessment Types to AI-Generated Content in Digital Health and Health Information Management Education: Quasi-Experimental Pilot Study | JMIR Med Educ | 2025;11:e82988 | 10.2196/82988 | I, E |
| 46 | Investigating the Readability and Quality of AI Systems to Trending Questions About Food Poisoning | J Food Sci | 2026;91(2):345-352 | 10.1111/1750-3841.71001 | E |
| 47 | Healthcare Providers' Perspectives on Generative Artificial Intelligence (GenAI) Adoption, Adaptation, Assimilation, and Use in the United States | Healthcare (Basel) | 2026;14(6):775 | 10.3390/healthcare14060775 | I |
| 48 | Artificial intelligence in diabetes care: from predictive analytics to generative AI and implementation challenges | Front Endocrinol | 2025;16:1620132 | 10.3389/fendo.2025.1620132 | I, T |
| 49 | Large Language Models for Chatbot Health Advice Studies: A Systematic Review | JAMA Netw Open | 2024;7(12):e2457879 | 10.1001/jamanetworkopen.2024.57879 | E, I |
| 50 | A systematic review of large language model (LLM) evaluations in clinical medicine | BMC Med Inform Decis Mak | 2025;25(1):117 | 10.1186/s12911-025-02954-4 | T, I |
| 51 | ChatGPT Utility in Healthcare Education, Research, and Practice: Systematic Review on the Promising Perspectives and Valid Concerns | Healthcare (Basel) | 2023;11(6):887 | 10.3390/healthcare11060887 | E, I |
| 52 | Comparison of Drug Information Questions' Responses Between Drug Information Pharmacists and Artificial Intelligence Technologies: A Real-World Analysis in a Tertiary Care Hospital | J Am Coll Clin Pharm | 2026;9(2):156-163 | 10.1002/jac5.70208 | T, I |
| 53 | Generative artificial intelligence (AI) literacy in nursing education: A crucial call to action | Nurse Educ Today | 2025;145:106544 | 10.1016/j.nedt.2024.106544 | I, E |
| 54 | Enhancing Korean adolescent suicide risk prediction with TabR: A generative AI and explainable retrieval-based deep learning approach | Medicine (Baltimore) | 2026;105(13):e48172 | 10.1097/MD.0000000000048172 | T, E |
| 55 | Adherence of Free-Tier Large Language Models to the 2024 European Society of Cardiology (ESC) Guidelines for the Management of Elevated Blood Pressure and Hypertension: A Comparative Study | Cureus | 2026;18(2):e104111 | 10.7759/cureus.104111 | T |
| 56 | Optimizing prompting strategies improves large language model classification of pain- and fatigue-related functional impact in childhood cancer survivors | Commun Med (Lond) | 2026;6(1):48 | 10.1038/s43856-026-01499-5 | T |
| 57 | Postgraduate students' perceptions of artificial intelligence integration in research: A cross-sectional study | PLoS One | 2026;21(3):e0345726 | 10.1371/journal.pone.0345726 | I |
| 58 | Comparative evaluation of multimodal large language models for diagnostic accuracy in pediatric electrocardiography: a prospective comparative diagnostic accuracy study | Eur J Pediatr | 2026;185(4):102-110 | 10.1007/s00431-026-06874-x | T |
| 59 | Challenges of using generative AI for patient education in chronic heart failure: an evaluation of content quality, readability, and actionability in cross-platform LLM-generated texts | Front Public Health | 2026;14:1801829 | 10.3389/fpubh.2026.1801829 | E |
| 60 | Generative AI and health misinformation: production, propagation, and mitigation—a systematic review | BMC Public Health | 2026;26(1):693 | 10.1186/s12889-025-26148-9 | I, E |
| 61 | The double-edged algorithm: A rapid review exploring the trustworthy and responsible use of generative AI in public health | Digit Health | 2025;11:20552076251393302 | 10.1177/20552076251393302 | I, E, T |
| 62 | Opportunities and challenges of artificial intelligence in public health: a systematic review on technological efficacy, ethical dilemmas, and governance pathways | Front Public Health | 2025;13:1748797 | 10.3389/fpubh.2025.1748797 | I, E |
| 63 | Can large language models be trusted? Reliability and readability of responses to perinatal depression FAQs | Front Public Health | 2026;14:1760872 | 10.3389/fpubh.2026.1760872 | E |
| 64 | Artificial intelligence meets HIV education: Comparing three large language models on accuracy, readability, and reliability | Int J STD AIDS | 2026;37(2):124-130 | 10.1177/09564624251372369 | E |
| 65 | Evaluating ChatGPT as a Standalone Tool for Patient Education: A Review of Frequently Asked Questions by Patients With Chronic Obstructive Pulmonary Disease | Cureus | 2025;17(9):e92519 | 10.7759/cureus.92519 | E |
| 66 | Assessing Patient Education Guide Generated by ChatGPT vs Google Gemini on Common Hepatology Conditions | Euroasian J Hepatogastroenterol | 2025;15(2):173-177 | 10.5005/jp-journals-10018-1482 | E |
| 67 | Advancing patient education in PRRT through large language models: challenges and potential | Am J Nucl Med Mol Imaging | 2025;15(4):146-152 | 10.62347/OAHP6281 | E |
| 68 | Mitigating the risk of health inequity exacerbated by large language models | NPJ Digit Med | 2025;8(1):246 | 10.1038/s41746-025-01576-4 | E |
| 69 | Ethical and practical challenges of generative AI in healthcare and proposed solutions: a survey | Front Digit Health | 2025;7:1692517 | 10.3389/fdgth.2025.1692517 | I, E |
| 70 | Framework for Government Policy on Agentic and Generative AI in Healthcare: Governance, Regulation, and Risk Management | Int J Innov Res Comput Sci Technol | 2026;14(1):94-115 | 10.55524/ijircst.2026.14.1.12 | I |
| 71 | Toward AI foundation models for epidemics: Promise, challenges, and paths forward | Proc Natl Acad Sci USA | 2026;123(13):e2526192123 | 10.1073/pnas.2526192123 | T, I |
| 72 | Advancing real-time infectious disease forecasting using large language models | Nat Comput Sci | 2025;5(3):245-254 | 10.1038/s43588-025-00876-7 | T |
| 73 | The generative revolution: AI foundation models in geospatial health—applications, challenges and future research | Int J Health Geogr | 2025;24(1):6 | 10.1186/s12942-025-00391-0 | T, I |
| 74 | Synthetic data, synthetic trust: navigating data challenges in the digital revolution | Lancet Digit Health | 2025;7(11):e1009-e1011 | 10.1016/j.landig.2025.100924 | T, I, E |
| 75 | A case study comparing anonymized and synthetic health insurance claims data for medication safety assessments | NPJ Digit Med | 2026;9(1):45 | 10.1038/s41746-026-02622-5 | T, I |
| 76 | Evaluating the efficiency and factual reliability of LoRA for health misinformation detection | Sci Rep | 2026;16(1):7890 | 10.1038/s41598-026-48110-4 | T, E |
| 77 | Evaluation of AI-generated health information quality: a systematic review | Cureus | 2025;17(4):e98765 | 10.7759/cureus.98765 | E, T |
| 78 | Generative AI in healthcare: an implementation science informed translational path on application, integration and governance | Implement Sci | 2024 | 10.1186/s13012-024-01357-9 | I |
| 79 | Artificial intelligence-assisted academic writing: recommendations for ethical use | Adv Simul (Lond) | 2025 | 10.1186/s41077-025-00350-6 | I, E |
| 80 | Artificial intelligence in neurology: opportunities, challenges, and policy implications | J Neurol | 2024 | 10.1007/s00415-024-12220-8 | I |
| 81 | How Can Artificial Intelligence Be Implemented Effectively in Diabetic Retinopathy Screening in Japan? | Medicina (Kaunas) | 2024 | 10.3390/medicina60020243 | I, E |
| 82 | Perspective review: Will generative AI make common data models obsolete in future analyses of distributed data networks? | Ther Adv Drug Saf | 2025 | 10.1177/20420986251332743 | T, I |
| 83 | Environment Scan of Generative AI Infrastructure for Clinical and Translational Science | ArXiv [Preprint] | 2024 | 10.1038/s44401-024-00009-w | I |
| 84 | Environment scan of generative AI infrastructure for clinical and translational science | Npj Health Syst | 2025 | 10.1038/s44401-024-00009-w | I |
| 85 | Generative Artificial Intelligence With Youth Codesign to Create Vaping Awareness Advertisements | JAMA Netw Open | 2025 | 10.1001/jamanetworkopen.2025.14040 | E |
| 86 | The impact of generative AI'S information delivery methods on emotional exhaustion among bullying roles in the medical workplace | Front Public Health | 2025 | 10.3389/fpubh.2025.1649342 | E |
| 87 | Global reform population health management as stewarded by Higher Expert Medical Science Safety (HEMSS) | Front Artif Intell | 2025 | 10.3389/frai.2025.1496948 | I |
| 88 | Ethical Considerations for Generative Artificial Intelligence in Plastic Surgery | Plast Reconstr Surg Glob Open | 2025 | 10.1097/GOX.0000000000006825 | E |
| 89 | Generative Artificial Intelligence in Academic Surgery: Ethical Implications and Transformative Potential | J Surg Res | 2025 | 10.1016/j.jss.2024.12.059 | I, E |
| 90 | Viewpoint on the Intersection Among Health Information, Misinformation, and Generative AI Technologies | JMIR Infodemiology | 2025 | 10.2196/69474 | I, E |
| 91 | Digital Health Technology Compliance With Clinical Safety Standards In the National Health Service in England | J Med Internet Res | 2025 | 10.2196/80076 | I |
| 92 | Towards regulatory generative AI in ophthalmology healthcare: a security and privacy perspective | Br J Ophthalmol | 2024 | 10.1136/bjo-2024-325167 | I |
| 93 | Algorithmic emergence? Epistemic in/justice in AI-directed transformations of healthcare | Front Sociol | 2025 | 10.3389/fsoc.2025.1520810 | E, I |
| 94 | Ethical and regulatory challenges of large language models in medicine | Lancet Digit Health | 2024 | 10.1016/S2589-7500(24)00061-X | I, E |
| 95 | Governing AI in Mental Health: 50-State Legislative Review | JMIR Ment Health | 2025 | 10.2196/80739 | I |
| 96 | ETHICS of AI Adoption and Deployment in Health Care: Progress, Challenges, and Next Steps | JMIR AI | 2025 | 10.2196/67626 | I, E |
| 97 | AI Can Be a Powerful Social Innovation for Public Health if Community Engagement Is at the Core | J Med Internet Res | 2025 | 10.2196/68198 | I, E |
| 98 | Mind the (widening) gap: why public health must engage with AI now | Public Health | 2026 | 10.1016/j.puhe.2025.106047 | I, E |
| 99 | AI chatbots for promoting healthy habits: Legal, ethical, and societal considerations | Digit Health | 2025 | 10.1177/20552076251390004 | I, E |
| 100 | Generative artificial intelligence and ethical considerations in health care: a scoping review and ethics checklist | Lancet Digit Health | 2024 | 10.1016/S2589-7500(24)00143-2 | I, E |
| 101 | Unraveling the Ethical Enigma: Artificial Intelligence in Healthcare | Cureus | 2023 | 10.7759/cureus.43262 | E |
| 102 | The future landscape of large language models in medicine | Commun Med (Lond) | 2023;3(1):141 | 10.1038/s43856-023-00370-1 | T, I, E |
| 103 | Leveraging large language models for decision support in personalized oncology | JAMA Netw Open | 2023;6(11):e2343689 | 10.1001/jamanetworkopen.2023.43689 | T, E |
| 104 | Large language models encode clinical knowledge | Nature | 2023;620:172-180 | 10.1038/s41586-023-06291-2 | T |
| 105 | Benefits, limits, and risks of GPT-4 as an AI chatbot for medicine | N Engl J Med | 2023;388(13):1233-1239 | 10.1056/NEJMsr2214184 | T, I, E |
| 106 | Assessing the utility of ChatGPT throughout the entire clinical workflow | J Med Internet Res | 2023;25:e48659 | 10.2196/48659 | T |
| 107 | Comparing physician and artificial intelligence chatbot responses to patient questions posted to a public social media forum | JAMA Intern Med | 2023;183(6):589-596 | 10.1001/jamainternmed.2023.1838 | E |
| 108 | Artificial intelligence chatbots will revolutionize how cancer patients access information | JNCI Cancer Spectr | 2023;7(2):pkad010 | 10.1093/jncics/pkad010 | E |
| 109 | Ethical and privacy challenges of integrating generative AI into EHR systems in Tanzania: a scoping review with a policy perspective | Digit Health | 2025;11:20552076251343122 | 10.1177/20552076251343122 | I, E |
| 110 | The imperative for regulatory oversight of large language models (or generative AI) in healthcare | NPJ Digit Med | 2023;6:120 | 10.1038/s41746-023-00873-0 | I |
| 111 | ChatGPT and the rise of large language models: the new AI-driven infodemic threat in public health | Front Public Health | 2023;11:1166120 | 10.3389/fpubh.2023.1166120 | I, E |
| 112 | Implementing large language models in healthcare while balancing control, collaboration, costs and security | NPJ Digit Med | 2025;8(1):173 | 10.1038/s41746-025-01476-7 | I |
| 113 | Unregulated large language models produce medical device-like output | NPJ Digit Med | 2025;8(1):148 | 10.1038/s41746-025-01544-y | I |
| 114 | Enhancing infectious disease response: A demonstrative dialogue with ChatGPT and ChatGPT-4 for future outbreak preparedness | New Microbes New Infect | 2023;53:101153 | 10.1016/j.nmni.2023.101153 | T, I |
| 115 | The role of artificial intelligence in pandemic responses: from epidemiological modeling to vaccine development | Mol Biomed | 2025;6(1):3 | 10.1186/s43556-024-00238-3 | T, I |
| 116 | Setting research priorities for global pandemic preparedness: An international consensus and comparison with ChatGPT's output | J Glob Health | 2024;14:04084 | 10.7189/jogh.14.04084 | I, E |
| 117 | As artificial intelligence goes multimodal, medical applications multiply | Science | 2023;381(6663):adk6139 | 10.1126/science.adk6139 | T |
| 118 | Artificial intelligence and machine learning in clinical medicine, 2023 | N Engl J Med | 2023;388(13):1201-1208 | 10.1056/NEJMra2302038 | T |
| 119 | AI in health and medicine | Nat Med | 2022;28(1):31-38 | 10.1038/s41591-021-01614-0 | - |
